# The Behaviour Change Technique Ontology: Transforming the Behaviour Change Technique Taxonomy v1

**DOI:** 10.12688/wellcomeopenres.19363.2

**Published:** 2024-05-09

**Authors:** Marta M. Marques, Alison J. Wright, Elizabeth Corker, Marie Johnston, Robert West, Janna Hastings, Lisa Zhang, Susan Michie

**Affiliations:** 1 Centre for Behaviour Change, University College London, London, England, UK; 2 Comprehensive Health Research Centre (CHRC), NOVA National School of Public Health, NOVA University of Lisbon, Lisbon, Lisbon, Portugal; 3 Institute of Pharmaceutical Science, King's College London, London, England, UK; 4 Clinical and Applied Psychology Unit, Department of Psychology, The University of Sheffield, Sheffield, England, UK; 5 Aberdeen Health Psychology Group, University of Aberdeen, Aberdeen, Scotland, UK; 6 Institute for Implementation Science in Health Care, Faculty of Medicine, University of Zurich, Zurich, Switzerland; 7 School of Medicine, University of St Gallen, St. Gallen, St. Gallen, Switzerland

**Keywords:** behaviour change techniques, ontology, user feedback, intervention reporting

## Abstract

**Background:**

The Behaviour Change Technique Taxonomy v1 (BCTTv1) specifies the potentially active content of behaviour change interventions. Evaluation of BCTTv1 showed the need to extend it into a formal ontology, improve its labels and definitions, add BCTs and subdivide existing BCTs. We aimed to develop a Behaviour Change Technique Ontology (BCTO) that would meet these needs.

**Methods:**

The BCTO was developed by: (1) collating and synthesising feedback from multiple sources; (2) extracting information from published studies and classification systems; (3) multiple iterations of reviewing and refining entities, and their labels, definitions and relationships; (4) refining the ontology via expert stakeholder review of its comprehensiveness and clarity; (5) testing whether researchers could reliably apply the ontology to identify BCTs in intervention reports; and (6) making it available online and creating a computer-readable version.

**Results:**

Initially there were 282 proposed changes to BCTTv1. Following first-round review, 19 BCTs were split into two or more BCTs, 27 new BCTs were added and 26 BCTs were moved into a different group, giving 161 BCTs hierarchically organised into 12 logically defined higher-level groups in up to five hierarchical levels. Following expert stakeholder review, the refined ontology had 247 BCTs hierarchically organised into 20 higher-level groups. Independent annotations of intervention evaluation reports by researchers familiar and unfamiliar with the ontology resulted in good levels of inter-rater reliability (0.82 and 0.79, respectively). Following revision informed by this exercise, 34 BCTs were added, resulting in the first published version of the BCTO containing 281 BCTs organised into 20 higher-level groups over five hierarchical levels.

**Discussion:**

The BCTO provides a standard terminology and comprehensive classification system for the content of behaviour change interventions that can be reliably used to describe interventions. The development and maintenance of an ontology is an iterative and ongoing process; no ontology is ever ‘finished’. The BCTO will continue to evolve and grow (e.g. new BCTs or improved definitions) as a result of user feedback and new available evidence.

## Introduction

Descriptions of ***behaviour change interventions*** vary widely, undermining the ability to synthesise evidence or replicate interventions for evaluation or implementation. This is a barrier to accumulating evidence about intervention effectiveness and thus making recommendations for research, policy, and practice. It also hinders developing more effective interventions. For this reason, a method for specifying intervention content was developed in the form of a structured ***taxonomy*** of behaviour change techniques, the Behaviour Change Technique Taxonomy v1 (BCTTv1) ( Michie *et al.*, 2013; Michie *et al.*, 2015). This paper describes the development and evaluation of the next generation of intervention description technology, the Behaviour Change Technique Ontology (BCTO).

***Behaviour change techniques (BCTs)*** are defined as the smallest parts of the content of a behaviour change intervention that are observable, replicable and on their own have the potential to bring about behaviour change ( Michie *et al.*, 2021a). The BCTTv1, developed with the input of 400 experts from around the world, comprises 93 BCTs, organised in 16 higher-order groupings based on cluster analysis of connections made by experts ( Michie *et al.*, 2013; Michie *et al.*, 2015). It provides a standardised, shared language to describe the ‘active ingredients’ of an intervention. Resources were developed to support the use of BCTTv1, including a smartphone app ( http://bit.ly/BCTsappGoogle; http://bit.ly/BCTsappApple), online training to guide the identification of BCTs in published papers ( http://www.bct-taxonomy.com/), a database of studies of interventions coded using BCTTv1 ( www.bct-taxonomy.com/interventions) and the Theory and Techniques Tool to link BCTs to their hypothesised mechanisms of action ( https://theoryandtechniquetool.humanbehaviourchange.org) ( Michie *et al.,* 2021a).

BCTTv1 has been widely applied internationally, reported in more than 5000 published studies. These cover intervention design and evaluation, evidence synthesis and implementation of behaviour change interventions in research and practical settings. Using meta-regression, it has been applied to investigate the effectiveness of individual or group-based behaviour change interventions across a wide range of populations, settings, and behaviours (e.g., Carraça *et al.*, 2021; Michie *et al.*, 2018). In combination with frameworks such as the Behaviour Change Wheel ( Michie *et al.*, 2011; Michie *et al.*, 2014), BCTTv1 has enabled a structured and systematic method for designing and evaluating interventions.

The BCTTv1 was intentionally named ‘v1’ to signal that developments to the taxonomy would be needed as the field advanced and feedback from users accumulated. To inform the improvement of the BCTTv1, we brought together user feedback from six sources ( Corker *et al.*, 2023). These were the BCT website, a user survey, researchers and experts involved in the Human Behaviour-Change Project, an interview-based consultation exercise of researchers and other users, relevant published research reports and other classification systems of BCTs. This feedback suggested a need to extend the BCTTv1, improve the labels and make the definitions more precise, and develop the structure to be more flexible, extensive, and multi-level.

Structures for representing knowledge by defining ***classes*** of ***entities*** (anything that exists in the universe) and their ***relations*** are called ***ontologies*** ( Arp *et al.*, 2015). In this paper, the terms ‘entity’ and ‘class’ are often used interchangeably to refer to entities represented in an ontology. For the definitions of technical terms used in this paper (in bold and italicised), see the glossary in Table 1. Entities and their relations are defined to represent their essential properties in such a way that they are uniquely and fully specified and assigned a unique label. This enables data to be computer-readable, and thus allows computational analysis of large amounts of complex data. ‘Computer-readable’ refers to a data structure that can be directly processed by computers, which is not possible for natural language text. Representation of information in computer-readable data structures allows computers to process information unambiguously and effortlessly. According to the ***Open Biological and Biomedical Ontology (OBO) Foundry*** principles of good practice for developing ontologies, ontologies must be made available in a common formal language in an accepted concrete syntax (Principle 2), which is typically the ***Web Ontology Language (OWL)***. This is necessary to investigate how behaviour change intervention components interact in producing effects and explanations of variation across, for example, populations, settings, and behaviours.

**Table 1.** Glossary of terms.

Term	Definition	Source
Annotation	Process of coding, or tagging, selected parts of documents or other resources to identify the presence of ontology entities.	Michie *et al.* (2017)
Annotation guidance manual	Written guidance on how to identify and tag pieces of text from intervention evaluation reports with specific codes relating to classes in the ontology, using for example EPPI-Reviewer software.	Michie *et al.* (2017)
Basic Formal Ontology (BFO)	An upper-level ontology specifying foundational distinctions between different types of entity, such as between continuants and occurrents, developed to support integration, especially of data obtained through scientific research.	Arp *et al.* (2015)
Behaviour Change Intervention (BCI)	An intervention that has the aim of influencing human behaviour.	Michie *et al.* (2021a)
Behaviour Change Intervention (BCI) content	A planned process that is part of a BCI and is intended to be causally active in influencing the outcome behaviour. Consists of at least one BCT. i.e. It is the active ingredients of interventions consisting of one or more behaviour change techniques.	Michie *et al.* (2021a)
Behaviour Change Technique (BCT)	The smallest parts of the content of a behaviour change intervention that are observable, replicable and on their own have the potential to bring about behaviour change	Michie *et al*. (2013)
Class	Classes in ontologies represent types of entities in the world. The terms “entity” and “class” are often used interchangeably to refer to the entities represented in an ontology. Classes can be arranged hierarchically by the specification of parent and child classes; see definition of parent class in the glossary.	Arp *et al.* (2015)
Entity	Anything that exists or can be imagined, including objects, processes, and their attributes. It Includes mental process, i.e., the process and content of cognitive representations, and emotions.	Arp *et al.* (2015)
EPPI-Reviewer (EPPI = Evidence for Policy & Practice Information Centre)	A web-based software program for managing and analysing data in all types of systematic review (meta-analysis, framework synthesis, thematic synthesis etc.) It manages references, stores PDF files and facilitates qualitative and quantitative analyses. It also has a facility to annotate published papers.	Thomas *et al.* (2010) EPPI-Reviewer 4: http://eppi.ioe.ac.uk/eppireviewer4/ EPPI-Reviewer Web Version: https://eppi.ioe.ac.uk/eppireviewer-web/
GitHub	A web-based platform used as a repository for sharing computer code, allowing version control.	https://github.com/
Inter-rater reliability	Statistical assessment of similarity and dissimilarity of coding between two or more coders. If inter-rater reliability is high this suggests that ontology class definitions and labels are being interpreted similarly by the coders.	Gwet (2014)
Interoperability	Two systems are interoperable to the extent that the data in each system can be used by the other system. Note: An ontology is interoperable with another ontology if it can be used together with the other ontology.	http://www.obofoundry.org/principles/fp-010-collaboration.html
Intervention source	A role played by a person, population or organisation that provides an intervention.	Michie *et al.* 2021b
Issue tracker	An online log for problems identified by users accessing and using an ontology.	BCIO issue tracker: https://github.com/HumanBehaviour ChangeProject/ontologies/issues
The Open Biological and Biomedical Ontology (OBO) Foundry	A collective of ontology developers who are committed to collaboration and adherence to shared principles. The mission of the OBO Foundry is to develop a family of interoperable ontologies that are both logically well-formed and scientifically accurate.	Smith *et al.* (2007); www.obofoundry.org/
OBO Foundry principles	Good practice principles of ontology development and maintenance intended as normative for OBO Foundry ontologies. Ontologies submitted to OBO Foundry are evaluated against them.	http://www.obofoundry.org/principles/fp-000-summary.html
Ontology	A standardised representational framework providing a set of entities for the consistent description (or “annotation” or “tagging”) of data and information across disciplinary and research community boundaries.	Arp *et al.* (2015)
Parent class	A class within an ontology that is hierarchically related to one or more child classes (subclasses) such that all members of the child class are also members of the parent class, and all properties of the parent class are also properties of the child class.	Arp *et al.* (2015)
Process	Something that takes place over time.	Arp *et al.* (2015)
Relation	The manner in which two entities are connected or linked.	
ROBOT	An automated command line tool for ontology workflows.	Jackson *et al.* (2019); http://robot.obolibrary.org
Taxonomy	A hierarchy consisting of terms denoting types (entities) which are linked by subtype relations.	Arp *et al.* (2015)
Uniform Resource Identifiers (URI)	A string of characters that unambiguously identifies an ontology or an individual entity within an ontology. Having URI identifiers is one of the OBO Foundry principles.	http://www.obofoundry.org/principles/fp-003-uris.html
Versioning	Ontologies that have been released are expected to change over time as they are developed and refined, leading to a series of different files. Consumers of ontologies must be able to specify exactly which ontology files they used to encode their data or build their applications and be able to retrieve unaltered copies of those files in perpetuity. Versioning is one of the OBO Foundry principles.	http://www.obofoundry.org/principles/fp-004-versioning.html
Web Ontology Language (OWL)	A formal language for describing ontologies. It provides methods to model classes of entities, how they relate to each other and the properties they have. OWL is designed to be interpreted by computer programs and is extensively used in the Semantic Web where rich knowledge about web documents and the relationships between them are represented using OWL syntax.	https://www.w3.org/TR/owl2-quick-reference/

Ontologies offer a more comprehensive and expressive way of representing information than taxonomies ( Hastings, 2017). For example, they can link BCTs to other intervention features such as their delivery, mechanisms of action and target behaviours, and context entities, such as population and setting. Ontologies also provide an effective method for connecting and accumulating knowledge across topic domains and academic disciplines (i.e. provide ‘ ***interoperability***’). Because they enable reporting in a clear, structured and transparent way, ontologies support clear communication and collaborative sharing of data between researchers and others. A further advantage of ontologies is that they are not static; they are designed to be added to and amended as new information accumulates from the use of the ontology and from scientific and intellectual advances.

**Figure 1.**
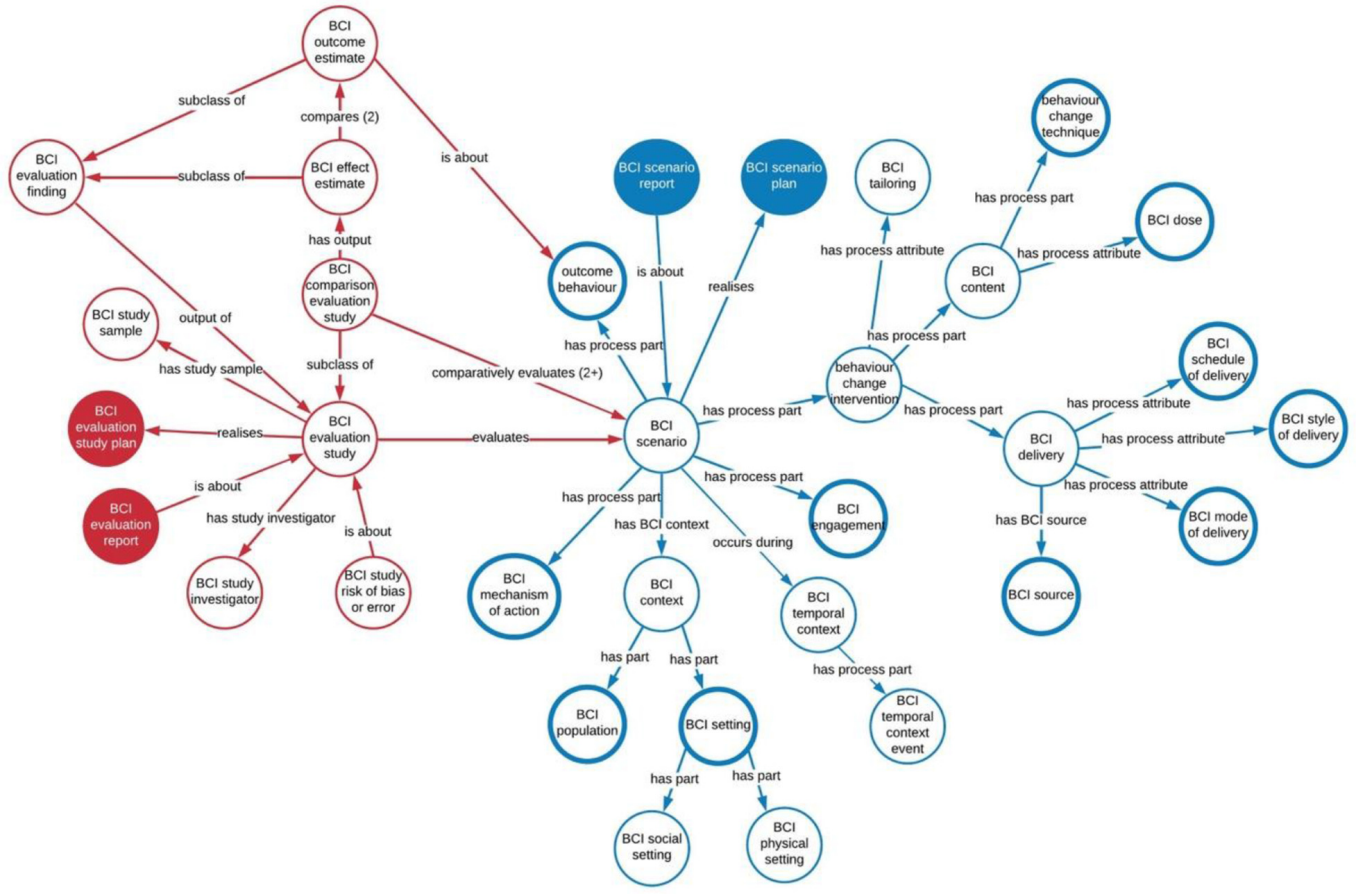
The upper level of the Behaviour Change Intervention Ontology (BCIO) V1.5. ‘Behaviour change technique’ is part of ‘BCI content’.

The development of a Behaviour Change Technique Ontology (BCTO) was informed by 282 feedback comments on BCTTv1 that suggested the need for additional BCTs, amendments to labels and definitions of specific BCTs, amendments to the groupings, and general improvements to increase clarity. The work was conducted as part of developing the overarching Behaviour Change Intervention Ontology ( Michie *et al*., 2021b), part of the Human Behaviour-Change Project ( Michie *et al.*, 2020). The Behaviour Change Intervention Ontology (BCIO) represents behaviour change interventions and their evaluations. The BCIO consists of an upper level with 42 entities, one of which is behaviour change intervention content ( ***BCI content***), which includes the entity “behaviour change technique” (see Figure 1). The BCIO covers the behaviour being targeted, how BCTs are delivered, e.g., their mode of delivery ( Marques *et al.*, 2021), schedule, style of delivery ( Wright *et al*., 2023), and source ( Norris *et al.*, 2021), the context (setting and population) in which they are delivered ( Norris *et al.*, 2020), and the mechanisms through which they produce behavioural changes ( Schenk *et al.*, 2023). Note that the *BCTO* is distinguished from the *BCIO* (i.e., the BCTO forms part of the larger BCIO).

The BCIO is intended to be used by both humans and computers. Humans can use the ontology to describe and report interventions, synthesise evidence (e.g. through literature annotation), and develop interventions, to name a few possible use cases. In computer-readable form, the ontology is represented in OWL, which can be parsed and used in several different downstream applications. For example, we can harness the power of computation in terms of Machine Learning and Artificial Intelligence to automate the extraction of information from reports ( West *et al*. 2023), and build systems that can predict behaviour in novel scenarios ( Hastings *et al*. 2023). Ontologies provide an interface between computers and humans, that is, computers can better support human tasks that require understanding of a domain, coupled with integration of content at scale and speed.

### Aim

This study aimed to develop an open-access, computer-readable Behaviour Change Technique Ontology (BCTO) that can be reliably used to describe the content of behaviour change interventions.

## Methods

### Ethical statement

Ethical approval was granted by the University College London's ethics committee (CEHP/2016/555).

### Design

The development of the Behaviour Change Technique Ontology (BCTO) consisted of six iterative steps based on methods developed in the Human Behaviour-Change Project ( Wright *et al*., 2020). Feedback from users of the BCTTv1 was analysed ( Corker *et al.*, 2023), BCT labels and definitions were rewritten to be consistent with ontological definitions ( Michie *et al*., 2019), BCTs were organised in a logical ontological structure, expert feedback was incorporated and ***inter-rater reliability*** of BCTs in annotated intervention reports was assessed.

### Step 1: Extract and synthesise feedback on the BCTTv1

Feedback about limitations and proposed improvements was collected from six sources: users of the BCTTv1 through the BCT website, a user survey, researchers and experts involved in the Human Behaviour-Change Project, an interview-basedconsultation of a purposive sample of global users, andrelevant published research reports and other classification systems. These data were analysed and synthesised to produce recommendations to inform the development of the BCT ontology (see Results section and Corker *et al*., 2023).

### Step 2: Changes to BCTs: labels and definitions

Step 1 recommendations were applied to each BCT label and definition, and changes made to aid clarity and specificity, where necessary, by authors EC and MJ. The revised BCTs were reviewed and amended where necessary by five of the study behavioural science experts (EC, MM, MJ, SM, and RW) and one ontology expert (JH).

### Step 3: Structuring the BCTO as an ontology

Step 1 recommendations were applied to each group label and BCTs within each group by EC and MM. Changes to add clarity were proposed by EC and MJ. The full set of revised group labels and BCTs within each group were reviewed and amended where necessary by the full team (EC, MM, MJ, SM, RW and JH). All BCTs were reviewed to ensure they had a hierarchical relationship ("is_a" in ontological terms) with their ***parent class*** (i.e., meaning the BCT is a subclass of the higher-level group it belongs to). New BCTs were discussed in relation to which group they belonged to by EC, MM, MJ and SM prior to the final team review to ensure that each group was inclusive (i.e., they contained BCTs with a common active content element) and exclusive (i.e., the BCTs within each group did not belong in any other group).

### Step 4: Expert stakeholder review

21 expert stakeholders (17 behavioural scientists and 4 ontology experts) reviewed the BCTO resulting from Step 3 so that the ontology reflected broader scientific consensus about BCTs as well as meeting the requirements of ontology users ( Wright *et al.*, 2020). Behavioural scientists were recruited from a database of those expressing willingness to participate as expert reviewers for studies conducted at the UCL Centre for Behaviour Change. To be eligible, participants were required to have a doctoral level degree in behavioural science or a related discipline. We excluded those who were close collaborators of the BCTO’s lead developers, i.e., had co-authored a publication in the previous three years or worked for the same institution. We purposively sampled to ensure geographical diversity and a range of career stages (from early career postdoctoral researchers to full professors or equivalent). Ontology experts were suggested by the Human Behaviour-Change Project’s ontology expert (JH). Prospective participants were sent an invitation and study information sheet. Those willing to participate in the study were sent a link to an online questionnaire ( https://osf.io/2gs9b) ( West *et al.*, 2020), along with the BCTO displayed in both spreadsheet and diagram forms. Experts were asked to review all groups of BCTs (and the individual BCTs within that group) one at a time, taking an estimated 2.5 hours and were paid an honorarium for doing this. The expert review was conducted using Qualtrics&#8482;.

Of the 17 behavioural scientists invited to participate, eight completed the review. In addition, two behavioural scientists developing a physical activity ontology provided feedback. Three of the four invited ontology experts completed the review. The 13 providing feedback worked in institutions based in the United Kingdom (n=4), Belgium (n=3), South Africa (n=1), Canada (n=1) and USA (n=4).

Participants were presented with the label of a single higher-level group from the BCTO and all the BCTs within that group. For each BCT, participants were asked to indicate whether any labels or definitions needed refining and, if so, to suggest alternatives. They were asked for additional BCTs and for any other comments about the BCTO. Following a conference presentation, we received feedback from the Habit Special Interest Group of the European Health Psychology Society on the seven BCTs that were at that point in the “habit BCT” group. All feedback was discussed by the research team and led to revising BCT labels or definitions, rearranging BCT groupings, removing or adding BCTs or providing explanations for not revising.

### Step 5: Inter-rater reliability of annotations using the BCTO

Inter-rater reliability of ***annotations*** using the BCTO was assessed in two ways. First, two researchers involved in the development of the BCTO independently annotated 50 papers from Cochrane reviews (25 on smoking cessation and 25 on physical activity). This number was selected as it gives a 10-15% margin of error around the estimated percentage agreement between coders ( Gwet, 2014; Wright *et al.*, 2020). Annotations followed an ***annotation guidance manual*** ( https://osf.io/mwv2c) ( West *et al.*, 2020). From this set of annotations, any necessary changes to the manual and labels or definitions of the BCTs were made. In the second assessment of inter-rater reliability, two behaviour change experts experienced in annotating behaviour change intervention reports but with no prior knowledge of the ontology, independently annotated a randomly selected sample of 50 randomised controlled trials from a database of papers coded using the Behaviour Change Techniques Taxonomy v1 ( http://www.bct-taxonomy.com/interventions).

The papers focused on the following target behaviours: physical activity (k=18), consumption behaviours (k=10), healthcare use and medication adherence (k=6), sexual health behaviours (k=6), multiple health promotion behaviours (k=5), hygiene behaviours (k=3) and smoking cessation (k=2). Annotations were conducted using ***EPPI-Reviewer 4*** software ( Thomas *et al.*, 2010). An open alternative to this software that can be used for annotations is PDFAnno ( Shindo *et al.*, 2018)].

Inter-rater reliability was assessed using Krippendorff’s Alpha ( Hayes & Krippendorff, 2007) calculated using version 1.0.0 of the Automation Inter-Rater Reliability script developed for the Human Behaviour-Change Project ( Finnerty & Moore, 2020). The research team made additional changes to the BCTO based on the issues arising from inter-rater reliability testing, as well as from a final revision of the consistency between ***class,*** definitions and labels.

### Step 6: Computer-readable version of the BCTO and publication in online repositories

The BCTO was developed as a table of entities, with separate rows for each ***entity*** and its label, definition, synonyms, examples, relationships with other entities, and elaboration. When the BCTO was at a stable level of development for the first release it was converted into the computable Web Ontology Language (OWL) ( Antoniou & Van Harmelen, 2004) format, which is a standard representation format for ontologies widely used across domains. The OWL representation of the ontology can be searched, visualised and queried using standard ontology tools and software. The conversion was done using the ***ROBOT*** ontology toolkit library ( Jackson *et al.*, 2019).

The OWL version of the BCTO is stored in the Human Behaviour-Change Project’s GitHub repository), an online platform for sharing and ***versioning*** resources. The ***GitHub*** repository has an ***issue tracker*** which allows feedback and queries to be submitted by members of the GitHub community; these can be responded to and, if necessary, addressed in subsequent ontology releases. The BCTO is part of the Behaviour Change Intervention Ontology which is available online in the Behavioural and Social Sciences Ontology Foundry, a repository for ontologies in the behavioural and social science domains; and an associated community of practice is being built. The final Behaviour Change Intervention Ontology will be submitted to the Open Biological and Biomedical Ontology (OBO) Foundry ( Smith *et al.*, 2007).

## Results

**Figure 2.**
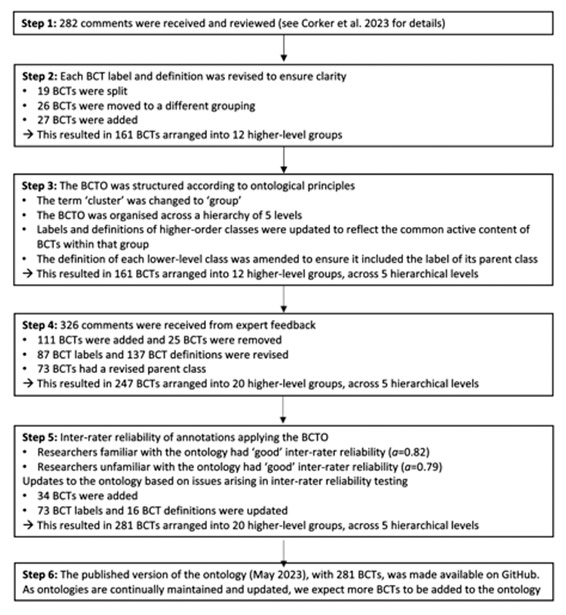
Summary of results of the steps for the BCTO development.

The results from each step of the BCTO development are presented in Figure 2 and described below.

### Step 1: Extract and synthesise feedback on the BCTTv1

A total of 282 comments from the feedback exercises and published reports were received and used for review. These were organized into four categories: i. 32 comments containing 47 suggestions for new BCTs, ii. 92 comments related to amendments to labels and definitions of specific BCTs, iii. 9 comments related to amendments to the groupings, and iv. 17 comments containing suggestions for general improvements. Changes resulting from these recommendations are provided in Steps 2 and 3 (for full details also see Corker *et al.*, 2023).

### Step 2: Changes to BCTs: labels and definitions

First, the definition of the term ‘behaviour change technique’ was updated to comply with ontological terms. The definition agreed by the research team was " *A planned **process** that is the smallest part of BCI content that is observable, replicable and on its own has the potential to bring about behaviour change*" ( Michie *et al.*, 2021a).

Second, each BCT was amended in the following ways:

- BCT labels were revised so that each clearly aligned to a specific BCT definition.
- Suggestions to split 19 BCTs into different parts were agreed, for example, the BCT ‘Goal setting (behaviour)’ was split into ‘Set behaviour goal BCT’ and ‘Agree behaviour goal BCT’ ( https://osf.io/j5wgb) ( West *et al.*, 2020).
- BCT labels and definitions were revised to ensure clarity of the active content, that is, exactly what process the BCT is describing.
- Technical and theory-specific language was removed from labels and definitions to allow for understanding across disciplines.
- 26 BCTs were moved to a group that better reflected the active content described. For example, ‘demonstration of the behaviour BCT’ was moved from the BCTTv1 cluster ‘comparison of behaviour’ to the BCTTv1 cluster ‘shaping knowledge’ ( https://osf.io/j5wgb) ( West *et al.*, 2020).
- 27 new BCTs were agreed. These consisted of suggestions for 22 new BCTs from Step 1 and a further five agreed on during review meetings. For example, ‘facilitate integration of behaviour goals BCT’ was added to the ‘goal directed BCT’ group ( https://osf.io/j5wgb) ( West *et al.*, 2020).

This step resulted in 161 BCTs (see https://osf.io/8x2zn) ( West *et al.*, 2020).

### Step 3: Structuring the BCTO as an ontology

- The numerical indicators of the groupings of BCTs were removed to better reflect ontological nomenclature – i.e., that the primary labels of classes in ontologies should not ordinarily contain numeric codes. Numeric codes are instead captured in the unique identifiers or where needed as associated annotations. For example, the unique identifier for ‘Set behaviour goal BCT’ is BCIO:007003.
- The BCTO was organised into a three-level classification hierarchy ( https://osf.io/3tekn) ( West *et al.*, 2020). An individual BCT (e.g., ‘Set behaviour goal BCT’) was classified at the lowest level of the hierarchy (i.e., Level 3), with its parent class one level up in the hierarchy (e.g., ‘Goal setting BCT’ at Level 2). The highest-level of the ontology (i.e., Level 1) contains the parent classes of BCTs that share a common active content (e.g., ‘Goal directed BCT’)
- All parent classes names were changed to better reflect the common active content described by each BCT within the group.
- A definition for each parent class was added to ensure clarity regarding the nature of the BCTs within the group.
- Four higher-level groups (comparison of outcomes, scheduled consequences, self-belief and covert learning) were removed as it was agreed that the active content described by the BCTs within these groups were set out clearly in definitions for other groups.
- 13 lower-level parent classes were added across six groups to aid specificity. For example, the lower-level parent classes ‘goal setting BCT’ and ‘goal implementing BCT’ were added to the higher-level group ‘goal directed BCT’ (see https://osf.io/a6bwf) ( West *et al.*, 2020).
- The start of each BCT definition was amended to ensure that the definition included the label of its parent class, for example, the start of the definition for ‘goal setting BCT’ is ‘a goal directed BCT that changes behaviour by….’, where ‘goal directed BCT’ is the label for the higher-level group in which ‘goal setting BCT’ is placed.

This process resulted in 12 higher-level groups that represent hierarchically organised BCTs (see https://osf.io/3tekn) ( West *et al.*, 2020).

### Step 4: Expert stakeholder review

The experts provided 326 comments on the ontology via the online survey. The EHPS Habit SIG made 11 recommendations regarding the eight BCTs in the “Habit BCT” group (see https://osf.io/2jwqz for responses to comments collected via the online survey and https://osf.io/6vhp4 for responses to the EHPS Habit SIG’s recommendations ( West *et al.*, 2020)).

In response to experts’ feedback that it was unclear whether the definition of a BCT applied to both self-enacted behaviour change and behaviour change interventions delivered by a separate ***intervention source*** (e.g., health care professional), the definition of a BCT was changed to “A planned process that is the smallest part of behaviour change intervention content that is observable, replicable and on its own has the potential to bring about behaviour change *in oneself or other people*” (added text italicised.) We also removed mention of “the intervention source” from most definitions to clarify that BCTs could be delivered either by others or self-enacted.

Feedback from the ontology experts led to “that changes behaviour” being removed from the first part of the definition of all BCTs. For example, “goal setting BCT”’s definition was revised from “A goal-directed BCT that changes behaviour through goal setting” to “A goal-directed BCT that sets goals”. This reflects a principle of ontological definitions that they should reflect what is always true about members of a class, and behaviour change techniques do not always lead to changes in behaviour. To make it clearer that BCTs were processes (i.e., things that take place over time), we updated BCT labels to include a verb where possible.

A number of stakeholder comments pointed out that many BCTs have more than one potential mechanism of action. Therefore, BCT groups where the only shared feature of the BCTs was their hypothesised mechanism of action (e.g., habit BCT group, personal resources BCT group) were regrouped according to the type of process involved in the BCT itself (e.g., “advise specific behaviour BCT” or “suggest different perspective on behaviour BCT” groups). In response to comments that some of the “creating consequences”, “reward” and “incentive” BCTs had confusing definitions, we revised the organisation of these BCTs and added a number of new BCTs where their absence had been noted.

Expert feedback led to 25 BCTs being removed from the ontology (e.g. ‘prompt problem solving’ was removed as it could not be distinguished from ‘problem solving BCT’) and 111 BCTs added (e.g. ‘set measurable behaviour goal BCT’ was added as a child class of ‘set behaviour goal BCT’, as not all goals are measurable). All 137 BCTs retained from the Step 3 ontology had revised definitions, 87 had revised labels (e.g. ‘graded tasks BCT’ was amended to ‘set graded tasks BCT’ to include the verb) and 73 had a revised parent class (e.g. ‘monitor emotional consequences BCT’ was moved from the ‘awareness of consequences BCT’ group to the ‘monitoring BCT’ group. Over half (47/73) of the changes of parent class reflected the BCT being moved to a different higher-level group. The revised version of the BCTO following expert review had 247 BCTs arranged into 20 higher-level groups organised over five hierarchical levels (see https://osf.io/escjk) ( West *et al.*, 2020).

### Step 5: Inter-rater reliability of annotations using the BCTO

Inter-rater reliability from the 50 papers annotated by those familiar with the ontology was *a*=0.82 (see https://osf.io/7nqvb) and *a*=0.79 (see https://osf.io/u7dxs ( West *et al.*, 2020) for the 50 papers annotated by researchers unfamiliar with the ontology ( Hayes & Krippendorff, 2007). These are considered good levels of inter-rater reliability.

Final revisions were made to the BCTO based on: a) issues raised by the annotators, b) revision of the BCTO by the research team on clarity and consistency of labels and definitions of the BCTs, and c) additional suggestions from behavioural scientists on BCTs that were missing or BCTs that still required more clarity. The following changes were made:

- 34 BCTs were added. For example, two child classes were created for ‘inform about health consequences BCT’, which were ‘inform about positive health consequences BCT’ and ‘inform about negative health consequences BCT’
- The labels of 73 BCTs were updated. For example, ‘advise how to perform behaviour BCT’ was changed to ‘guide how to perform behaviour BCT’
- The definitions of 16 BCTs were updated.

### Step 6: Computer-readable version of the BCTO and publication in online repositories

**Figure 3.**
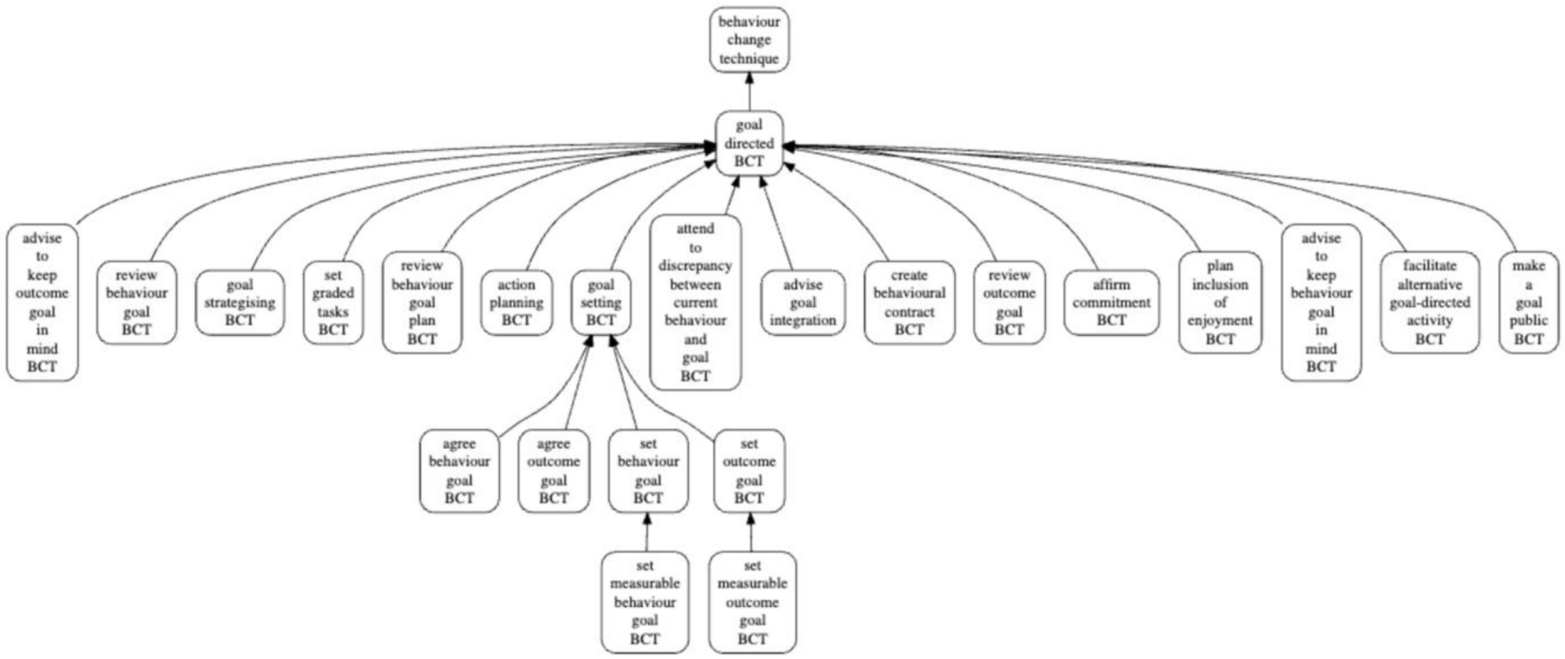
Snapshot of the hierarchical structure of the “goal directed BCT” group.

The first release of the BCTO consisted of 281 BCTs hierarchically organised into 20 higher-level groups, with between one and 77 BCTs per higher-level group. The 20 higher-level BCT groups, their definitions and number of BCTs per group are shown in Table 2. An excerpt from BCTO showing the BCTs belonging to the higher-level group "goal directed BCT" is shown in Table 3. To facilitate visualisation, Figure 3 shows the hierarchical relationships between entities in the “goal directed BCT” group, using the dedicated online tool, BCIOVisualise.

**Table 2.** Definitions of the 20 higher-level groups in the final BCT Ontology, and number of BCTs in each group (after step 5).

BCT group	Definition	No. of BCTs in this group
**Goal directed BCT**	A behaviour change technique that sets or changes goals.	23
**Monitoring BCT**	A BCT that involves gathering or using information about performance.	12
**Social support BCT**	A BCT that involves taking steps to secure or deliver the support or aid of another person.	16
**Guide how to perform behaviour BCT**	A BCT that provides guidance regarding how to perform the behaviour.	6
**Conduct a behavioural experiment BCT**	A BCT that advises on how to identify and test hypotheses about the behaviour, its causes and consequences.	1
**Suggest different perspective on** **behaviour BCT**	A BCT that suggests the deliberate adoption of a new perspective on the behaviour.	5
**Increase awareness of behaviour BCT**	A BCT that draws attention to the behaviour.	3
**Increase awareness of consequences BCT**	A BCT that draws attention to consequences of the behaviour in the normal course of events.	21
**Awareness of other people’s thoughts,** **feelings or actions BCT**	A behaviour change technique that increases awareness of what other people think, do, or feel.	7
**Associative learning BCT**	A behaviour change technique that involves repeated pairing of a stimulus with another stimulus or with a behavioural outcome.	15
**Advise specific behaviour BCT**	A behaviour change technique that advises the person to perform a behaviour in a particular way to help change the target behaviour.	9
**Manage mental processes BCT**	A behaviour change technique that advises how to manage mental processes to facilitate the target behaviour.	4
**Prompt thinking related to successful** **performance BCT**	A behaviour change technique that prompts thinking relating to successful performance of a behaviour.	6
**Change the body BCT**	A behaviour change technique that alters the structure or functioning of the person's body.	1
**Promote pharmacological support BCT**	A behaviour change technique promoting medicines or other drugs.	3
**Advise how to change emotions BCT**	A BCT that suggests a method to alter emotions.	20
**Restructure the environment BCT**	A behaviour change technique that alters the environment in which the behaviour is, or would have been, performed in a way that facilitates or impedes the behaviour.	12
**Prompt focus on self-identity BCT**	A BCT that prompts the person to focus on their mental representation of themself.	5
**Behavioural consequence BCT**	A behaviour change technique that alters the consequences or promised consequences for a behaviour.	77
**Outcome consequence BCT**	A behaviour change technique that alters the consequences or promised consequences for an outcome that results from performing or not performing a behaviour.	35

*Note.* The number of BCTs in each of group should not be considered final. The BCTO will continue to evolve and grow (e.g. new BCTs or new groups) as a result of user feedback and new available evidence.

**Table 3.** Excerpt from the BCTO presenting the ‘goal directed BCT’ group.

Level 1 label	Level 2 label	Level 3 label	Level 4 label	Definition
goal directed BCT BCIO:007001				A <behaviour change technique> that sets or changes goals.
	goal setting BCT BCIO:007002			A <goal directed BCT> that sets goals.
		set behaviour goal BCT BCIO:007003		A <goal setting BCT> that sets a goal for the behaviour to be achieved.
			set measurable behaviour goal BCT BCIO:007300	A <set behaviour goal BCT> that describes the behaviour to be achieved in terms of a measurable target.
		agree behaviour goal BCT BCIO:007004		A <goal setting BCT> that involves the intervention source agreeing with the person on a behavioural goal.
		set outcome goal BCT BCIO:007005		A <goal setting BCT> in which the goal is a positive outcome of performing the behaviour.
			set measurable outcome goal BCT BCIO:007300	A <set outcome goal BCT> that describes the behavioural outcome to be achieved in terms of a measurable target.
		agree outcome goal BCT BCIO:007006		A <goal setting BCT> that involves the intervention source agreeing with the person on a goal which is a positive outcome of performing the behaviour.
	make a goal public BCT BCIO:007122			A <goal directed BCT> that involves the person communicating their intention to achieve a goal.
	set graded tasks BCT BCIO:007100			A <goal directed BCT> that sets easy-to-perform tasks for the person, making them increasingly difficult, but achievable, until the behaviour is performed.
	goal strategising BCT BCIO:007008			A <goal directed BCT> in which the person analyses factors influencing the behaviour and generates, selects, or reviews strategies to increase facilitators and overcome barriers.
	action planning BCT BCIO:007010			A <goal directed BCT> that involves making a detailed plan for the performance of the behaviour, which must include at least one of context, frequency, duration or intensity.
	review behaviour goal BCT BCIO:007011			A <goal directed BCT> that reviews a behavioural goal and considers modifying the goal in light of progress toward the goal.
	review behaviour goal plan BCT BCIO:007299			A <goal directed BCT> that reviews progress towards a behavioural goal and considers modifying the plan to achieve the goal.
	attend to discrepancy between current behaviour and goal BCT BCIO:007012			A <goal directed BCT> that draws attention to discrepancies between a person’s current behaviour and the person’s outcome goal, behavioural goal or action plan.
	review outcome goal BCT BCIO:007013			A <goal directed BCT> that reviews an outcome goal and considers modifying the goal in light of achievement.
	create behavioural contract BCT BCIO:007014			A <goal directed BCT> that creates a written specification of the behaviour to be performed, agreed on by the person, and witnessed by another person.
	affirm commitment BCT BCIO:007015			A <goal directed BCT> that asks the person to affirm or reaffirm statements indicating commitment to change the behaviour.
	facilitate alternative goal- directed activity BCT BCIO:007171			A <goal directed BCT> that enables the person to engage in an alternative rewarding behaviour that increases the likelihood of goal achievement.
	advise goal integration BCT BCIO:007016			A <goal directed BCT> that advises the person to adopt more than one goal in a situation where the goals operate synergistically.
	plan inclusion of enjoyment BCT BCIO:007061			A <goal directed BCT> that advises the person to plan a way of performing the behaviour that is pleasurable or satisfying.
	advise to keep behaviour goal in mind BCT BCIO:007141			A <goal directed BCT> that advises the person to find ways to remind themselves of their behavioural goal.
	advise to keep outcome goal in mind BCT BCIO:007142			A <goal directed BCT> that advises the person to find ways to remind themselves of their goal in terms of a desired outcome of performing the behaviour.

*Note.* The BCTO forms part of the larger BCIO, therefore all entity URIs hold the form BCIO:XXXXXX *.* The BCTO will continue to evolve and grow as a result of user feedback and new available evidence, so it is likely that new BCTs will be added to this group or definitions will be updated.

A downloadable version of the BCTO is available from GitHub ( https://github.com/HumanBehaviourChangeProject/ontologies/tree/master/BehaviourChangeTechniques/inputs) and it can be browsed in the dedicated BCIOSearch tool and the Ontology Lookup Service. The hierarchical structure, ***Uniform Resource Identifiers (URI)***, labels and definitions for all entities are described in https://osf.io/ya74q ( West *et al.*, 2020). The ontology is accompanied by an annotation guidance manual on how to annotate entities in behaviour change intervention reports ( https://osf.io/mwv2c ( West *et al.*, 2020)).

Some important general observations and clarifications about the BCTO that emerged during the development process are outlined in Box 1. They concern delivery of BCTs by self or others, differences between a new BCT and instances of the same BCT, preparatory BCTs and combinations of BCTs.

**Box 1. Clarifications about the BCTO.**

**Clarifications about the BCTO**

**1. Most BCTs can be delivered by an external intervention provider or self-initiated.**

Most BCT definitions have been written in such a way that they can apply a) when the BCT is delivered by someone other than the person whose behaviour is being targeted, b) when the BCT is delivered without a person acting as the intervention source (e.g., in digital or print interventions), or c) when a BCT is self-enacted or initiated as part of a behaviour change attempt.

The exception to this rule is when the involvement of an intervention source as well as the person changing the target behaviour is inherent to the nature of the BCT. Examples of this include ‘agree on behavioural goal BCT’ and ‘agree on outcome goal BCT’, as an agreement needs to be made with another, ‘create behavioural contract BCT’ as part of this BCT is having the contract witnessed by another and ‘observation of behaviour by another without feedback BCT’. In such cases, the definitions of the BCTs make clear that another person has to be involved.

**2. Difference between a new BCT and instances of the same BCT.**

When reading authors’ descriptions of their interventions or when classifying BCTs during intervention development or evaluation, one may notice what appears to be a new BCT. BCTs can be added to the ontology when it is confirmed that they can be defined in a manner that differentiates them from the BCTs already included in the ontology. In most cases, these apparently new BCTs turn out to be particular implementations or child classes of BCTs that are already in the ontology. Examples or the former are different solutions that can be used to overcome barriers to a given behaviour, or variations of action planning. Different child classes of a BCT can be added to the ontology if their specific features are important.

**3. Preparatory behaviours**

Behaviours that are required for a target behaviour to be performed are not behaviour change techniques *per se*. For example, obtaining a prescription for a medicine to aid smoking cessation is a requirement for taking the medication.

**4. Combinations of BCTs**

Some BCTs are often delivered alongside other BCTs. However, for simplicity and to avoid making the BCTO larger, we have not included classes representing combinations of BCTs because it would be impossible to include all the combinations of BCTs that intervention developers might view as important. Some intervention classification systems that we reviewed included categories that are combinations of BCTs. For example, in social prescribing, “connect to social support” is an intervention strategy ( Cunningham *et al*. 2022). Examination of the definition of “connect to social support” revealed that it was a combination of two BCTs (advise to seek social support BCT followed by arrange social support BCT). Therefore, we have not included “connect to support” as a BCT. However, given the importance of this strategy in social prescribing, we have noted in the elaboration of “arrange social support BCT” and “advise social support BCT” that when used together, they may be termed “connect to support”. When it applies, logically defined classes of BCT combinations can be added.

**5. Advantages of BCTO over BCTTv1**

Ontological systems may be specified in various ways (such as controlled lists, thesauri, taxonomies, or formal representations in logic), and these lie on a continuum of semantic complexity ( National Academies of Sciences, Engineering, and Medicine, 2022). Formal ontologies (e.g., BCTO) include strong semantics (specifying properties of entities and their relations in formal logic), whereas taxonomies (e.g. BCTTv1) include weak semantics (may only specify parent-child relations).

Readers may ask why they should consider using BCTO over BCTTv1 (given the latter is widely used) and there are many reasons for this. Feedback from multiple sources (see Corker *et al.*, 2023 for details) on BCTTv1showed: (i) the need to add more BCTs (more than 93 BCTs exist), (ii) the cluster labels from BCTTv1 were undefined and difficult to understand, and (iii) the taxonomical structure of BCTTv1 was based on cluster analyses, therefore not logical and did not allow the addition of BCTs. The BCTO has several advantages including: (i) being more complete than BCTTv1, (ii) having a logical structure (BCTs can be added), (iii) more precise and clear groupings, labels and definitions, (iv) links to other aspects of an intervention scenario, such as mechanisms of action, and (v) being computer-readable, which can support the application of artificial intelligence and machine learning approaches in data extraction, evidence synthesis, and outcome prediction.

## Discussion

This study has developed a logically structured Behaviour Change Technique Ontology for describing and classifying BCTs using a computer-readable common terminology. The first published version consists of 281 BCTs organised into 20 higher-level groups and five hierarchical levels. It is published on an open-source platform alongside tools for visualisation and searching ( https://github.com/HumanBehaviourChangeProject/ontologies/tree/master/BehaviourChangeTechniques).

The BCTO is part of the Behaviour Change Intervention Ontology, currently made up of 11 ontologies: intervention delivery mode ( Marques *et al.*, 2021), source ( Norris *et al.*, 2021), schedule and dose (in preparation), style ( Wright *et al*., 2023), human behaviour (in preparation), mechanisms of action ( Schenk *et al.*, 2023), engagement (in preparation), fidelity (in preparation), and contextual influences such as intervention setting ( Norris *et al.*, 2020) and target population ( Michie *et al*., 2020). The BCTO allows one to represent interventions in their contexts in a comprehensive and structured way enabling the answering of complex questions along the lines of: “When it comes to behaviour change interventions: What works, compared with what, for what behaviours, how well, for how long, with whom, in what setting, and why?” Answering variants of this question requires large quantities of data and sophisticated analyses; it requires automation and the application of Artificial Intelligence to identify relevant studies, extract relevant information and organise it within an ontology so that predictions can be made, drawing on the full range of intervention and contextual features. This was the aim of, and was mostly achieved, by the Human Behaviour-Change Project ( Michie *et al.*, 2020; https://www.humanbehaviourchange.org); it represents a step-change in the potential to accumulate evidence to address complex behavioural questions, thereby improving theories of behaviour change, the development of more effective interventions and the science of behaviour change more generally.

This improved method of specifying behavioural intervention content overcomes some limitations of BCTTv1 but will continue to need updating and improving.

### Key differences between the BCTO and the Behaviour Change Technique Taxonomy v1 (BCTTv1)

The BCTO contains considerably more BCTs and classes than BCTTv1. It also has a deeper hierarchical structure. In BCTTv1, the BCTs were organised over two levels. In the BCTO, BCTs are organised in a five-level hierarchy. The BCTO’s hierarchy provides a more logical organisation of classes. For example, in the BCTTv1, “social support (unspecified)” and “social support (emotional)” are on the same level, even though emotional social support is a particular type of general social support. In the BCTO, “advise to seek emotional support BCT” is a child class of the parent class “advise to seek support BCT.” The position of a BCT in the hierarchy reflects the organisation of the BCTO according to ontological principles. BCTs at the deeper levels of the hierarchy are more granular and specific than those at higher levels of the hierarchy.

The vast majority of BCTs from BCTTv1 can be mapped to one or more BCT in the BCTO (see https://osf.io/r7cux; West *et al.*, 2020). In BCTTv1, the labels of groups were selected based on the group’s content and where applicable, the frequency of words in labels provided by participants ( Michie *et al.*, 2013). BCTO labels reflect good practice in writing labels for ontology classes ( Michie *et al.*, 2019). Some BCTTv1 groups were labelled according to the hypothesised mechanism of action of the BCTs in that group (e.g. “shaping knowledge” or “self-belief”). In the BCTO, BCTs or their classes were not generally defined in terms of their potential mechanisms of action, because many BCTs can have more than one mechanism of action, depending on context, how the BCT is delivered, or which other BCTs are delivered at the same time. An Ontology of Mechanisms of Action ( Schenk *et al.*, 2023), also part of the Behaviour Change Intervention Ontology, can be used in conjunction with BCTO to describe both intervention content in terms of BCTs and their hypothesised mechanisms of action. The mapping from BCTTv1 to the BCTO ( https://osf.io/r7cux; West *et al.*, 2020), will be useful to those wishing to link up information classified by BCTTv1 with that classified by BCTO and those using BCTO whilst who also wish to use the Theory and Techniques Tool, an evidence-based method for linking BCTs with their hypothesised mechanisms of action ( Johnston *et al.*, 2021). We have also produced the ‘reverse’ mapping i.e. mapping BCTO to BCTTv1, so users can see all BCTs, including which are new and which can be mapped by back to BCTs in BCTTv1 (see https://osf.io/ru3q2).

Definitions of BCTs in BCTO conform to principles for writing “good” ontological definitions ( Michie *et al.*, 2019; Seppälä *et al.*, 2017). Each definition describes a BCT in terms of its parent class plus the things that differentiate the BCT from its parent class. To fully understand the nature of a BCT from its definition, ontology users will need to check the definition of the parent class stated in the first part of the definition. The structure of BCTTv1 was derived from a cluster analysis of experts’ groupings of BCTs. As a result, it was not possible to add new BCTs to a group without repeating the expert grouping task and statistical analyses. Additionally, for some groups, there was no clear unifying feature of the BCTs in the group. The logical structure of BCTO overcomes these problems by having an explicit basis for the inclusion of BCTs within a group. Moreover, because the BCTO has a logically defined structure, new BCTs that are identified can be added to the ontology where they fit best, based on their ontological definitions. Future changes in the ontology can be recorded, along with explicit reasons for the changes.

### Strengths and limitations

A strength of this study is the use of a standardised, tried and tested method for ontology development created within the Human Behaviour-Change Project ( Wright *et al*., 2020) that reflects the ***OBO Foundry principles*** of good practice in ontology development ( Norris *et al*., 2019; Smith *et al*., 2007). As a result, the BCTO fares well against several ontology evaluation criteria ( Vrandečić, 2009). The BCTO’s *completeness,* or how well it covers the domain of interest, was tackled by extending BCTTv1 and using scoping terms from other BCT classification systems. Completeness was also checked as part of the expert review, with experts asked if they thought any BCTs were missing from the ontology. The ontology’s *accuracy,* in terms of how well it accords with experts’ knowledge, was addressed by having the ontology developed by a team of researchers with considerable expertise in behaviour change interventions and by subjecting the ontology to expert review. The ontology’s *clarity,* in other words whether it communicates the intended meaning of the defined classes, was examined through inter-rater reliability testing, which assesses whether independent annotators can agree on what constitutes an example of a BCT, using the definitions in the ontology.

A strength of the development of the BCTO is the use of international expert feedback in revising the ontology, a practice which has been uncommon in ontology development ( Norris *et al.*, 2019). Involving a range of experts provides a variety of perspectives on the ontology which is necessary to build consensus around definitions. Participating experts were recruited via social media and newsletter dissemination by the UCL Centre for Behaviour Change and by the Human Behaviour-Change Project. Ontology experts were recruited by the team’s ontology expert. While the ontology benefits from the incorporation of international expert stakeholder feedback in the ontology development process, only half of invited behavioural science experts provided feedback on the ontology. With more time and resources, other means of engaging participants from under-represented parts of the world and disciplines could have been developed and are likely to have led to wider representation. Our aim is to disseminate the BCTO, and the Behaviour Change Intervention Ontology more generally, as widely as possible, to engage users and encourage feedback as the ontology is used in a variety of settings and for a variety of purposes.

### The status and future of the BCTO

Ontologies should be maintained and updated according to new evidence about entities and relationships ( Arp *et al.*, 2015; He *et al.*, 2018). The development and maintenance of an ontology is an iterative process; no ontology is ever ‘finished’. This is the first published version of the BCTO and it will continue to evolve. As with other ontologies produced as part of the Human Behaviour-Change Project ( Michie *et al.*, 2020), the BCTO will be refined through application and feedback from users via GitHub ( https://github.com/HumanBehaviourChangeProject/ontologies/issues). For instance, if users believe additional entities are needed in the ontology, or if a BCT definition should be revised, then they make these suggestions on GitHub and these will be reviewed by the ontology developers. Guidance on how to do this can be found on the project website ( https://www.bciontology.org/contribute). If the suggestions are appropriate (as discussed and decided among the research team or through further stakeholder feedback), the development team will make the changes. User suggestions and subsequent changes will be made on a periodic basis (e.g., every 6 months) and we will explicitly document responses to user suggestions and changes between released versions of the ontology. Normally, ontologies are maintained for at least three years from the time of acceptance into the OBO Foundry. Collaborations formed through the BSSO Foundry ( https://www.bssofoundry.org/) – a foundry for ontologies in behavioural and social sciences – can also support the evolution and maintenance of the BCTO. This commitment to ongoing updates and revisions to the BCTO creates opportunities for feedback from a broad range of experts to enhance and elaborate the ontology. The scope of the BCTO and associated ontologies within the Behaviour Change Intervention Ontology was limited to a level of generality considered to be of wide interest and use; those focussing on specific parts in more detail will need to extend the ontology.

It is also important that users report the unique identifier (e.g. BCIO: XXXXXX) in their intervention descriptions, so it is clear as exactly to which BCT(s) were used. Whilst entity labels and definitions can change over time (e.g. based on user feedback or new scientific evidence), the unique identifier always remains the same. Furthermore, as the content of ontologies can change over time, we also recommend that ontology users report the publication date of the ontology version that they used in their work.

The BCTO is designed to be connected (“interoperable”) not only with the other parts of the Behaviour Change Intervention Ontology but also with ontologies in other fields, for example, health care, neuroscience, mental functioning, research methods and biology. It achieves this through the use of the standard ontology representation language, OWL, community-agreed metadata standards, and a common framework for the upper-level structure of ontologies, the “ ***Basic Formal Ontology***” (BFO; Arp *et al.*, 2015; Grenon & Smith, 2004; Smith & Grenon, 2004). BFO’s upper-level structure divides things that exist in the world into two overarching categories: “continuants”, which are objects and spatial entities that continue to exist as the same individual over time, such as an intervention’s geographical setting, and “occurrents”, which are events or processes that unfold in time. All the BCTs in the BCTO are processes. The BCTO’s interoperability with ontologies from related fields creates exciting potential for future cross-disciplinary working and data integration.

The BCTO provides an improved method of specifying behavioural intervention content that overcomes identified limitations of the Behaviour Change Techniques Taxonomy v1; it will continue to need updating and improving. In the future, updated versions of the BCT Ontology will be released via GitHub [ https://github.com/HumanBehaviourChangeProject/ontologies/tree/master/BehaviourChangeTechniques] and all updates will be available in the BCIOSearch and OLS tools as well as via the Behavioural and Social Sciences Ontology Foundry repository. We recommend that prospective users of the ontology check these online resources to ensure they have the most recent version of the ontology. Training in using the BCIO has been developed as part of the Human Behaviour-Change Project, covering purposes such as describing interventions and their contexts, supporting intervention development and evaluation, structuring evidence reviews and sharing knowledge across disciplinary and domain boundaries ( www.bciontology.org/training).

The BCTO development method can be a useful resource to support other researchers in developing new ontologies in other areas (e.g., the Behaviour Change Intervention Ontology is being extended in relation to mental health interventions, see https://galenos.org.uk/) and in transforming existing classifications systems into ontologies. There is future work to be done in evaluating the BCTO as a resource for behavioural and social scientists and researchers; for intervention development and evaluation, this includes its added value for identifying BCTs that are most appropriate for given behaviours, context, delivery and mechanisms of action.

## Conclusion

The BCT Ontology provides a common terminology and comprehensive structure for describing and classifying BCTs that can enable more efficient evidence accumulation and synthesis about ‘what works’ in behaviour change interventions across scientific disciplines and behavioural domains. This improved method of specifying behavioural intervention content extends and improves the Behaviour Change Techniques Taxonomy v1 but will continue to need updating and improving in an ongoing and collaborative process. The ontology is being published on an open-source platform alongside tools for visualisation and searching alongside other ontologies that form the Behaviour Change Intervention Ontology, providing a foundation on which future research on behaviour change can build on.

## Consent

All participants provided their informed consent to participate in the study. The consent was obtained electronically through Qualtrics, the platform used for the survey. The participants indicated their consent by ticking a box. This consent process was in the ethics approval.

## Data Availability

Open Science Framework: Human Behaviour-Change Project. https://doi.org/10.17605/OSF.IO/EFP4X ( West *et al.*, 2020). The BCIO is available from: https://github.com/HumanBehaviourChangeProject/ontologies. Archived version of the ontology as at time of publication: https://github.com/HumanBehaviourChangeProject/ontologies/tree/master/BehaviourChangeTechniques/inputs Open Science Framework: Human Behaviour-Change Project. https://doi.org/10.17605/OSF.IO/EFP4X ( West *et al.*, 2020) This project contains the following extended data: Expert stakeholder feedback survey; Full survey provided to behavioural science and ontology experts in review of the BCTO; https://osf.io/2gs9b Annotation guidance; Manual for annotating using the BCTO; https://osf.io/mwv2c Summary of BCTs that were split, BCTs moved to a different higher-level group, and new BCTs added (step 2); https://osf.io/j5wgb New higher-level groups and parent classes of Behaviour Change Techniques (BCTs) (step 3); https://osf.io/a6bwf Version 0.1 of the Behaviour Change Technique Ontology; https://osf.io/8x2zn Version 0.2 of the Behaviour Change Technique Ontology; https://osf.io/3tekn Version 0.3 of the Behaviour Change Technique Ontology; https://osf.io/escjk Expert stakeholder feedback on BCTO; Raw feedback received from behavioural science and ontology experts, and responses from BCTO research team; https://osf.io/2jwqz Recommendations from the EHPS Habit SIG, and responses from BCTO research team; https://osf.io/6vhp4 Internal inter-rater reliability testing; https://osf.io/7nqvb External inter-rater reliability testing; https://osf.io/u7dxs First release of the Behaviour Change Technique Ontology (version at Step 6); https://osf.io/ya74q Mapping of BCTTv1 to the BCTO; https://osf.io/r7cux Mapping of BCTO to BCTTv1; https://osf.io/ru3q2 Data are available under the terms of the Creative Commons Attribution 4.0 International license (CC-BY 4.0).

## Software availability

Source code used to calculate alpha for IRR available from: https://github.com/HumanBehaviourChangeProject/Automation-InterRater-Reliability.

Archived code at time of publication: https://doi.org/10.5281/zenodo.3833816 ( Finnerty & Moore, 2020)

License: GNU General Public License v3.0
