# Supplementary material for: The Behaviour Change Technique Ontology: Transforming the Behaviour Change Technique Taxonomy v1
Source: Wellcome Open Res. 2024 May 9;8:308. Originally published 2023 Jul 17. [Version 2] doi: 10.12688/wellcomeopenres.19363.2 (PMC10427801; doi:10.12688/wellcomeopenres.19363.2)
Supplement: Peer review history

# Reviewer report: Reviewer response for version 1

**DOI:** 10.21956/wellcomeopenres.21449.r63996

**Published:** 2023-08-21

**Authors:** Judith Dyson

**Affiliations:** 1 Department of Health, Education and Life Sciences, Birmingham City University, Birmingham, England, UK

Here the authors seek to develop a behaviour change technique ontology (BCTO) through a six step process: 1. feedback on the BCTT1 from website and user survey; 2. author led subsequent changes; 3. author led BCTO structuring; 4. stakeholder review; 5. inter-rater reliability of BCTO use on published papers first by authors then two experts and; 6. machine readable version.

 Thank you for asking me to review this work, I enjoyed reading it. There is a great need for such an ontology for both researchers and practitioners. This work potentially makes a behaviour change approach more accessible to non-specialist/non-psychologist researchers and health care practitioners.

 The methods demonstrate a robust process and these were clear to me. I wondered why only four out of 17 invited behavioural scientist stakeholders participated and the in step 4 and the implications of this. Particularly as this small number of participants had so many comments. I wondered if a larger group may have been wise. That said, the definitions appeared to be effective enough for the assessors in step 5. From the discussion I understood the purpose, but I could not quite grasp the *output* of the machine-readable stage. I look forward to subsequent publications of this valuable work.

Is the work clearly and accurately presented and does it cite the current literature?

Yes

If applicable, is the statistical analysis and its interpretation appropriate?

Yes

Are all the source data underlying the results available to ensure full reproducibility?

Yes

Is the study design appropriate and is the work technically sound?

Yes

Are the conclusions drawn adequately supported by the results?

Yes

Are sufficient details of methods and analysis provided to allow replication by others?

Yes

Reviewer Expertise:

Implementation Science

I confirm that I have read this submission and believe that I have an appropriate level of expertise to confirm that it is of an acceptable scientific standard.

# Response

**Published:** 2024-04-08

**Authors:** Marta Marques

**Affiliations:** NOVA National School of Public Health, Lisbon, Portugal

Thank you for your positive feedback to our paper. All comments have now been addressed.

 The methods demonstrate a robust process and these were clear to me. I wondered why only four out of 17 invited behavioural scientist stakeholders participated and the in step 4 and the implications of this. Particularly as this small number of participants had so many comments. I wondered if a larger group may have been wise. That said, the definitions appeared to be effective enough for the assessors in step 5. Response: Eight out of the 17 invited behavioural scientists participated. We agree that feedback from a larger group of experts might have led to a greater range of suggested changes to the ontology, and this indeed may be a limitation of the study. Therefore, we have added a sentence on this in the “Strengths and limitations” section. From the discussion I understood the purpose, but I could not quite grasp the *output* of the machine-readable stage. I look forward to subsequent publications of this valuable work. Response: **We have added to the last paragraph of the introduction a description of how the BCTO (and wider BCIO) is intended to be used by both humans and computers (the output): *“The BCIO is intended to be used by both humans and computers. Humans can use the ontology to describe and report interventions, synthesise evidence (e.g. through literature annotation), and develop interventions, to name a few possible use cases. In computer-readable form, the ontology is represented in the Web Ontology Language (OWL) which can be parsed and used in several different downstream applications. For example, we can harness the power of computation in terms of Machine Learning and Artificial Intelligence to automate the extraction of information from reports (West et al. 2023), and build systems that can predict behaviour in novel scenarios (Hastings et al. 2023). Ontologies provide an interface between computers and humans, that is, computers can better support human tasks that require understanding of a domain, coupled with integration of content at scale and speed.”*

# Reviewer report: Reviewer response for version 1

**DOI:** 10.21956/wellcomeopenres.21449.r63997

**Published:** 2023-08-15

**Authors:** Emily Finne

**Affiliations:** 1 School of Public Health, Bielefeld University, Bielefeld, Germany

Thank you for inviting me to review this important paper on the further development of the most comprehensive classification system of behaviour change techniques.

 The article describes the further development of the BCT Taxonomy v1 into an ontology, inter-operable with other ontologies.  It describes all steps of this process and its results in a reproducible way.

 Overall, the paper is clearly written and conveys a complex content in a comprehensible way. The development steps from BCTTv1 to BCTO were described in a comprehensible way and sources for documented interim results as well as additional resources were linked.

 My comments and suggestions are mainly minor and aim at a better comprehensibility for interested people without prior knowledge of AI or computer science and new users who want to work with the "BCTO".

**Introduction:**

- In the 5th paragraph it would be helpful to make the differences between taxonomy and ontology a bit clearer (in my understanding mainly hierarchy versus network structure). Perhaps it should also be made clear first that this is a computer science term related to Artificial Intelligence and its application to texts. This would help to clarify some formal requirements and rules of ontologies.

*Last paragraph of introduction: *

- for clarity please add that the feedback comments were ***on BCTTv1.***
- When first mentioning the BCI ontology it may be useful to emphasise "Intervention"(for example writing it in italics) to distinguish it from BCTO. Since the work describes a complex development process related to many other pieces of work from the Human Behaviour Change project it may sometimes be hard for readers not familiar with the project to distinguish between all components, especially as they share integral constituents within their names and many similar abbreviations are used.

- In this context, I would appreciate it if a graphical overview of the project could be given, in which the parts named in the text (HBCP, BCIO, and the different ontologies on the same level as BCTO) are illustrated and shown in their connections, so that it is easier to classify them when reading. Such a diagram could replace the current Figure 1, as this essentially contains the headings to the design steps from the text and thus no additional information is given.

**Objective:**

 The aim of the work is clearly stated. Again, for readers who are not very familiar with AI tools, it might be useful to briefly explain what computer- or machine-readable means exactly, what requirements the ontology must fulfil for this, and to what extent the BCTO is intended to be used by humans and/or computer applications.

**Glossary:**

- My impression with the glossary was that some terms defined here are rather less important for the BCTO itself than for the overall project.  I wonder whether more technical entries on e.g. GitHub, versioning, OBO Foundry principles or ROBOT should actually be listed here. The latter, for example, occurs quite far back in the text and could simply be explained briefly by a half-sentence there.

- On the one hand, these are certainly terms that many readers will not know and therefore it can be useful to explain them in the glossary. On the other hand, they do not always seem necessary to me for understanding the ontology and its structure (e.g. the specific software or websites). It could also make sense to divide the glossary into more content-related ontology principles and terms versus more technical terms related to the tools used.

- The terms "class" and "entity" are defined differently but used interchangeably. This is somewhat confusing. For example, the definition of "entity" also refers to "classes" in addition to processes and attributes. It would improve readability if, at least in the text, a uniform term were predominantly used. 
- In the entry on "GitHub", it could be added that "code" refers to computer code.
- The entry on OWL talks about "things". I think one could also speak of classes or entities here, so as not to use yet another term?
- Not all of the terms explained can be found in the text. For example, "OBO Foundry Principles" are not mentioned in the text. These principles could also be added to the main entry on OBO. "Issue tracker" is highlighted in the text but not in the glossary.

**Design:**

 Step 4: Under this step it is first described that all experts should review all BCT groups. Then it says "Participants were presented with the label of a single higher-level group from the BCTO and all the BCTs within that group." - Should this mean "one group at a time" or "one after the other"? Otherwise it seems contradictory.

**Results:**

 I really much appreciate the extensive documentation of each step through additional online files linked in the text.

**Discussion:**

- The reference to a document with the mapping between BCTTv1 and BCTO annotations is very helpful. Overall, it seems important to me that intervention descriptions made on the basis of older versions can be assigned as clearly as possible to the current BCTO version, because otherwise part of the problem remains with different terms used for the same techniques.
- It would be helpful for potential users to describe more clearly how an update of the annotations for translations into the latest BCTO version can look in the future. This also seems important to me precisely because the BCTO can develop further and thus change.

**Future prospect:**

 "Future changes in the ontology can be recorded, along with explicit reasons for the changes." Keeping the ontology up to date, checking feedback and changing or adding entries if necessary will require a lot of work. Also, because different ontologies refer to each other. But it seems to me an important prerequisite to benefit from the ontology system in the best possible way, because some problems may only emerge over time with practical application.

 "...will continue to need updating and improving in an ongoing and collaborative process."

- Here it seems helpful to me to communicate current considerations in more detail. It is possible that procedures will also result from the principles of the open source network, but a few aspects do not seem clear enough to me and maybe others:
- Should the project continue as an open science project in which all interested users can generally participate? Will a qualified core team continue to be responsible for the adaptations? Above all: how will it be decided whether entries should be changed, according to which criteria and who can make changes? After all, an ontology that changes too frequently can no longer be used uniformly and be consensual.

- The link to the BCIO main page contains a typo and therefore does not work: Please remove superfluous "o" from www.bcio ***o*** ntology.org/training

Is the work clearly and accurately presented and does it cite the current literature?

Yes

If applicable, is the statistical analysis and its interpretation appropriate?

Not applicable

Are all the source data underlying the results available to ensure full reproducibility?

Yes

Is the study design appropriate and is the work technically sound?

Yes

Are the conclusions drawn adequately supported by the results?

Yes

Are sufficient details of methods and analysis provided to allow replication by others?

Yes

Reviewer Expertise:

Health Psychology, Public Health, Behaviour Change

I confirm that I have read this submission and believe that I have an appropriate level of expertise to confirm that it is of an acceptable scientific standard.

# Response

**Published:** 2024-04-08

**Authors:** Marta Marques

**Affiliations:** NOVA National School of Public Health, Lisbon, Portugal

Thank your for your positive feedback on our paper. All comments have been adressed and answered bellow. 

**Introduction:**

- In the 5th paragraph it would be helpful to make the differences between taxonomy and ontology a bit clearer (in my understanding mainly hierarchy versus network structure). Perhaps it should also be made clear first that this is a computer science term related to Artificial Intelligence and its application to texts. This would help to clarify some formal requirements and rules of ontologies.

Response: We have added a definition of taxonomy in the Glossary, and have also added to Box 1 the following points: (i) the difference between a taxonomy and ontology, (ii) why an ontology is needed even though BCTTv1 exists, and (iii) advantages of BCTO over BCTTv1. *Last paragraph of introduction:*

- for clarity please add that the feedback comments were ***on BCTTv1.***

Response: We have now added this.

- When first mentioning the BCI ontology it may be useful to emphasise "Intervention"(for example writing it in italics) to distinguish it from BCTO. Since the work describes a complex development process related to many other pieces of work from the Human Behaviour Change project it may sometimes be hard for readers not familiar with the project to distinguish between all components, especially as they share integral constituents within their names and many similar abbreviations are used.

Response: We have now written a brief note at the end of the last paragraph of the introduction to highlight that the BCTO is distinguished from the Behaviour Change Intervention Ontology (BCIO).

- In this context, I would appreciate it if a graphical overview of the project could be given, in which the parts named in the text (HBCP, BCIO, and the different ontologies on the same level as BCTO) are illustrated and shown in their connections, so that it is easier to classify them when reading. Such a diagram could replace the current Figure 1, as this essentially contains the headings to the design steps from the text and thus no additional information is given.

Response: We have removed Figure 1 to reduce redundancy, as the steps are detailed in-text. We have now added a diagram in the introduction of the “upper level BCIO”, which represents the key entities and causal connections in the BCIO, including the BCTO and other ontologies within BCIO. This new diagram is now Figure 1. **Objective:**

 The aim of the work is clearly stated. Again, for readers who are not very familiar with AI tools, it might be useful to briefly explain what computer- or machine-readable means exactly, what requirements the ontology must fulfil for this, and to what extent the BCTO is intended to be used by humans and/or computer applications. Response: To avoid any confusion, we have changed all instances of the term “machine-readable” to “computer-readable”, and have added an explanation of what we mean by “computer-readable” at the end of paragraph 5 in the introduction: *“‘Computer-readable’ refers to a data structure that can be directly processed by computers, which is not possible for natural language text. Representation of information in computer-readable data structures allows computers to process information unambiguously and effortlessly. According to the Open Biological and Biomedical Ontology (OBO) Foundry principles of good practice for developing ontologies, ontologies must be made available in a common formal language in an accepted concrete syntax (Principle 2), which is typically the Web Ontology Language (OWL).”* We have also added to the last paragraph of the introduction a description of how the BCTO (and wider BCIO) is intended to be used by both humans and computers: *“The BCIO is intended to be used by both humans and computers. Humans can use the ontology to describe and report interventions, synthesise evidence (e.g. through literature annotation), and develop interventions, to name a few possible use cases. In computer-readable form, the ontology is represented in OWL which can be parsed and used in several different downstream applications. For example, we can harness the power of computation in terms of Machine Learning and Artificial Intelligence to automate the extraction of information from reports (West et al. 2023), and build systems that can predict behaviour in novel scenarios (Hastings et al. 2023). Ontologies provide an interface between computers and humans, that is, computers can better support human tasks that require understanding of a domain, coupled with integration of content at scale and speed.”*

**Glossary:**

- My impression with the glossary was that some terms defined here are rather less important for the BCTO itself than for the overall project.  I wonder whether more technical entries on e.g. GitHub, versioning, OBO Foundry principles or ROBOT should actually be listed here. The latter, for example, occurs quite far back in the text and could simply be explained briefly by a half-sentence there.

Response: The glossary is intended to include low frequency terms or terms that may be unfamiliar to a behavioural and social science audience (especially terms relating to ontologies). Additionally, previous feedback from users have suggested that these technical terms were necessarily to include, as there was unfamiliarity with these terms. Therefore, we have decided to keep these entries in.

- On the one hand, these are certainly terms that many readers will not know and therefore it can be useful to explain them in the glossary. On the other hand, they do not always seem necessary to me for understanding the ontology and its structure (e.g. the specific software or websites). It could also make sense to divide the glossary into more content-related ontology principles and terms versus more technical terms related to the tools used.

Response: To make the glossary most user-friendly, we have decided to have one glossary that orders the terms in alphabetical form. As it is a somewhat challenging task to separate terms into ‘ontology principles’, ‘tools’, and so on, we will not divide the glossary. The terms "class" and "entity" are defined differently but used interchangeably. This is somewhat confusing. For example, the definition of "entity" also refers to "classes" in addition to processes and attributes. It would improve readability if, at least in the text, a uniform term were predominantly used.  Response: Classes in ontologies represent types of entities in the world. The terms ‘entity’ and ‘class’ are often used interchangeably in the literature to refer to entities represented in an ontology. To avoid confusion, we have amended the fifth paragraph of the introduction to be more precise. In the entry on "GitHub", it could be added that "code" refers to computer code. Response: We have now added this.

- The entry on OWL talks about "things". I think one could also speak of classes or entities here, so as not to use yet another term?

Response: We have now changed “things” to “entities”.

- Not all of the terms explained can be found in the text. For example, "OBO Foundry Principles" are not mentioned in the text. These principles could also be added to the main entry on OBO. "Issue tracker" is highlighted in the text but not in the glossary.

Response: We have added a sentence in the Discussion that include “OBO Foundry Principles” in the text, and have added “Issue tracker” in the glossary table. **Design:**

 Step 4: Under this step it is first described that all experts should review all BCT groups. Then it says "Participants were presented with the label of a single higher-level group from the BCTO and all the BCTs within that group." - Should this mean "one group at a time" or "one after the other"? Otherwise it seems contradictory. Response: Participants were asked to review BCT groups (and the BCTs within that group) one at a time. We have clarified this in the text in Step 4.

**Results:**

 I really much appreciate the extensive documentation of each step through additional online files linked in the text. Response: Thank you for your comments.

**Discussion:**

- The reference to a document with the mapping between BCTTv1 and BCTO annotations is very helpful. Overall, it seems important to me that intervention descriptions made on the basis of older versions can be assigned as clearly as possible to the current BCTO version, because otherwise part of the problem remains with different terms used for the same techniques.

Response: We agree that it is important that authors reporting intervention descriptions based on BCTTv1 might also consider mapping BCTs (from BCTTv1) to BCTO, using the mapping document. We have added a few sentences in the Discussion (“The status and future of the BCTO” section) to address the issue of reporting which version of the ontology authors use: *“* *It is also important that users report the unique identifier (e.g. BCIO: XXXXXX) in their intervention descriptions, so it is clear as exactly to which BCT(s) were used. Whilst entity labels and definitions can change over time (e.g. based on user feedback or new scientific evidence), the unique identifier always remains the same. Furthermore, as the content of ontologies can change over time, we also recommend that ontology users report the publication date of the ontology version that they used to their work.”*

- It would be helpful for potential users to describe more clearly how an update of the annotations for translations into the latest BCTO version can look in the future. This also seems important to me precisely because the BCTO can develop further and thus change.

Response: Thank you for raising this important issue. We have added to the section (“The status and future of the BCTO”) to address this issue: *“* *Ontologies should be maintained and updated according to new evidence about entities and relationships (* *Arp et al., 2015* *;* *He et al., 2018* *). The development and maintenance of an ontology is an iterative process; no ontology is ever ‘finished’.* *This is the first published version of the BCTO and it will continue to evolve.* *As with other ontologies produced as part of the Human Behaviour-Change Project (* *Michie et al., 2020* *), the BCTO will be refined through application and feedback from users via GitHub (* *https://github.com/HumanBehaviourChangeProject/ontologies/issues* *). For instance, if users believe additional entities are needed in the ontology, or if a BCT definition should be revised, then they make these suggestions on GitHub and these will be reviewed by the ontology developers. Guidance on how to do this can be found on the project website (* *https://www.bciontology.org/* *contribute* *). If the suggestions are appropriate (as discussed and decided among the research team or through further stakeholder feedback), the development team will make the changes. User suggestions and subsequent changes will be made on a periodic basis (e.g., every 6 months) and we will explicitly document changes between released versions of the ontology. Normally, ontologies are maintained for at least three years from the time of acceptance into the OBO Foundry.* *Collaborations formed through the BSSO Foundry (* *https://www.bssofoundry.org/* *) – a foundry for ontologies in behavioural and social sciences – can also support the evolution and maintenance of the BCTO.”* **Future prospect:**

 "Future changes in the ontology can be recorded, along with explicit reasons for the changes." Keeping the ontology up to date, checking feedback and changing or adding entries if necessary will require a lot of work. Also, because different ontologies refer to each other. But it seems to me an important prerequisite to benefit from the ontology system in the best possible way, because some problems may only emerge over time with practical application.

 "...will continue to need updating and improving in an ongoing and collaborative process."

- Here it seems helpful to me to communicate current considerations in more detail. It is possible that procedures will also result from the principles of the open source network, but a few aspects do not seem clear enough to me and maybe others:
- Should the project continue as an open science project in which all interested users can generally participate? Will a qualified core team continue to be responsible for the adaptations? Above all: how will it be decided whether entries should be changed, according to which criteria and who can make changes? After all, an ontology that changes too frequently can no longer be used uniformly and be consensual.

Response: The ontology indeed needs to reflect changes in scientific consensus to remain accurate over time. Updating and maintaining the ontology is a key principle in the OBO Foundry best practice for developing ontologies. The ontology will continue to evolve as an “open science project” in the sense that anyone can suggest changes through the “issue tracker” portal of Github. At least one member of the development team will be responsible for reviewing these user suggestions, and these will be discussed and decided among the research team or through further stakeholder feedback. Current team members are committed to doing this for at least the next six years. We will also ensure that the responsibility is handed over to others with the necessary expertise after this time period. As you rightly mentioned, ontology maintenance requires a lot of work, therefore, reviews and changes by the development team would occur on a periodic basis (e.g. every six months during the initial years of use, then less frequently such as yearly). Normally, ontologies are maintained for at least three years from the time of acceptance into the OBO Foundry. Changes will only be made if absolutely necessary – there must be good reason for these changes (e.g. new scientific evidence coming to light; a majority consensus from multiple users).

- The link to the BCIO main page contains a typo and therefore does not work: Please remove superfluous "o" from www.bcio ***o*** ntology.org/training

Response: Thank you for noticing the typo. It has now been corrected.

# Reviewer report: Reviewer response for version 1

**DOI:** 10.21956/wellcomeopenres.21449.r63730

**Published:** 2023-08-15

**Authors:** Tracy Epton

**Affiliations:** 1 Manchester Centre for Health Psychology, Division of Psychology and Mental Health, School of Health Sciences, Faculty of Biological, Medical and Health Sciences, The University of Manchester, Manchester, England, UK

The manuscript describes the development of a Behaviour Change Technique Ontology building on their former Behaviour Change Technique Taxonomy. The work described moves beyond the taxonomy of Behaviour Change Techniques (BCT) by refining existing and adding further BCTs, and situating the BCTs in an ontological framework to clarify the BCT properties and the relationships between the BCTs.

 The manuscript describes in detail a large, rigorously conducted, body of research that was used to achieve the current BCT Ontology (BCTO). Supplementary materials are provided to evidence all parts of the development process. There is also a process to update the BCTO that will futureproof this work. This BCTO will certainly benefit the science of behaviour change (and I’m excited to start using it!).

 I do have a few minor comments:

 Table 1

- Has some missing terms e.g., taxonomy, BCT
- Has some acronyms not spelled out e.g., BCI, EPPI

Methods

- In Step 1 there is a lack of detail about how many participants took part in the various aspects (it’s not clear from the text that this was the 282 comments mentioned in the introduction and Figure 2)
- Although the first three levels in the hierarchy are explained there is no explanation of what levels 4 and 5 are
- Examples throughout would be really useful. Where provided they make things a lot clearer without having to access the supplementary materials

Is the work clearly and accurately presented and does it cite the current literature?

Yes

If applicable, is the statistical analysis and its interpretation appropriate?

Not applicable

Are all the source data underlying the results available to ensure full reproducibility?

Yes

Is the study design appropriate and is the work technically sound?

Yes

Are the conclusions drawn adequately supported by the results?

Yes

Are sufficient details of methods and analysis provided to allow replication by others?

Yes

Reviewer Expertise:

Behaviour Change Interventions; Behaviour Change Techniques

I confirm that I have read this submission and believe that I have an appropriate level of expertise to confirm that it is of an acceptable scientific standard.

# Response

**Published:** 2024-04-08

**Authors:** Marta Marques

**Affiliations:** NOVA National School of Public Health, Lisbon, Portugal

The manuscript describes the development of a Behaviour Change Technique Ontology building on their former Behaviour Change Technique Taxonomy. The work described moves beyond the taxonomy of Behaviour Change Techniques (BCT) by refining existing and adding further BCTs, and situating the BCTs in an ontological framework to clarify the BCT properties and the relationships between the BCTs.

 We thank the reviewer for their very positive feedback about this paper. All comments made by the reviewer have been addressed in the manuscript and answered bellow. 

 Table 1: a) Has some missing terms e.g., taxonomy, BCT; b) Has some acronyms not spelled out e.g., BCI, EPPI Response: Both ‘taxonomy’ and ‘behaviour change technique (BCT)’ have been added to the glossary. We have also spelled out the acronyms. Methods

- In Step 1 there is a lack of detail about how many participants took part in the various aspects (it’s not clear from the text that this was the 282 comments mentioned in the introduction and Figure 2)

Response: We have added more information to step 1 in the methods section. However, it is important to reinforce that the comments result not only from consultation with users and experts but also from published papers, so they don’t correspond only to a sample of participants.

- Although the first three levels in the hierarchy are explained there is no explanation of what levels 4 and 5 are

Response: The three levels referred to an initial version of the BCTO, that was then expanded to 5 hierarchical levels. Levels correspond to parent and child classes, and this structure is explained in the results section.

- Examples throughout would be really useful. Where provided they make things a lot clearer without having to access the supplementary materials

Response: We have now provided examples in steps 2-5, as suggested. We hope these added examples are helpful to show how BCTs were transformed from BCTTv1 to BCTO.

# Reviewer report: Reviewer response for version 1

**DOI:** 10.21956/wellcomeopenres.21449.r63998

**Published:** 2023-08-07

**Authors:** Alexander K Saeri

**Affiliations:** 1 BehaviourWorks Australia, Monash Sustainable Development Institute, Monash University, Clayton, Victoria, Australia

The authors describe the research process to construct the Behaviour Change Technique Ontology (BCTO), an evolution of the widely used Behaviour Change Technique Taxonomy (BCTT), which describes more than 200 techniques that can change human behaviour, and the relationships between them. The article summarises and integrates six major sequential research activities described in detail in previously published work, which lead to an evidence and expert-informed ontology that can be used by researchers and machines to precisely define the content of behaviour change interventions, with an aim to improving future behaviour change research.

 I commend the authors on their rigorous and comprehensive approach to improving behaviour change research. This article provides a helpful overview of the research journey from the BCTT to the BCTO. I was generally able to follow along with the authors as they described gathering feedback on the BCTT; updating entity labels and descriptions; creating hierarchical relationships between entities to define the ontology; soliciting expert feedback; validating coding with interrater reliability; and publication. There were a couple of occasions where the balance of detail and clarity made it hard to understand exactly what happened at each step; I point these out below.

 Overall I thought this article describes an extremely strong contribution to behaviour change research. I have several **minor recommendations**, which are offered as collegial suggestions only; if the authors have a different preference I encourage them to not implement them if they feel it will not improve the value of the paper.

**1. Figures**: Figure 1 and Figure 2 could be combined, because they describe the same process. Figure 2 was somewhat inconsistent in its presentation; e.g., sometimes the number of BCTs at the end of each step was mentioned, and sometimes it was omitted. Figure 2 had some minor spelling errors (e.g., emended instead of amended).

**2. Examples**: when authors provided examples, it was much easier to understand each element of the process from initial BCTT to final BCTO. However, the inclusion of examples was inconsistent, e.g., for Step 2, an example was provided where 19 BCTs were split, but no other examples were provided for the other actions at this step. It did seem as though the goal directed BCTs was used throughout the process as an example where they were relevant, including in Table 3, but when the goal directed BCTs were not relevant no example was provided.

**3. Table 1 - Glossary**: I appreciate the inclusion of a glossary to assist readers in unfamiliar terms, but I was confused by the terms that were included (and omitted) from the glossary. For example, the elements that comprise an ontology were included (e.g., entity, class, relationship, ontology), but also common software tools/platforms (e.g., EPPI-Reviewer and GitHub). I didn’t understand why both ‘class’ and ‘parent class’ were included. ‘Ontology’ was defined, but ‘taxonomy’ was omitted. Finally, the term ‘BCI content’ was defined, but the acronym BCI was not spelled out in the definition. I suggest a review of the glossary to ensure it is as useful as possible for readers.

**4. Table 3 - Excerpt from the BCTO**: I thought this excerpt helped to communicate the hierarchical relationship between the entities in the ontology. However, I could imagine it being even more useful if presented as a hierarchical figure, such as a tree diagram. This would more explicitly show the relationships between entities (e.g., through branches on the tree). A separate comment for Table 3 is that the inclusion of the BCIO codes (e.g., BCIO:007001) is somewhat confusing because that the table purports to be an excerpt of the BCTO, not the BCIO. In the Discussion, I do see that the BCTO will form part of the Behaviour Change Intervention Ontology (BCIO), so all BCTO entities would therefore have a BCIO code. However, it may assist readers include a clarifying note for the table, or omit the BCIO code.

**5. Clarifications for non-expert readers**: the use of Box 1 to answer or clarify some questions that arose during the development process for the BCTO was very helpful. Two other clarifications - already described in text but useful to highlight - could clarify questions for interested, non-expert readers.

- The first clarification is about the difference between a taxonomy and an ontology, and why an ontology was needed even though the BCTT already exists and is widely used. This is already addressed in the introduction, but making this comparison more prominent could help a reader who knows about the BCTT, but not the BCTO and its advantages.
- The second clarification is about the difference between a behaviour change *technique *and a behaviour change *intervention*. This is mentioned several times in the introduction (e.g., “Behaviour change techniques are defined as…” and later “ontologies… can link BCTs to other intervention features such as their delivery…”) but again, making this comparison more prominent could help a reader understand how BCTs ‘fit’ within an intervention, and how the BCTO fits with the larger BCIO.

Is the work clearly and accurately presented and does it cite the current literature?

Yes

If applicable, is the statistical analysis and its interpretation appropriate?

Not applicable

Are all the source data underlying the results available to ensure full reproducibility?

Yes

Is the study design appropriate and is the work technically sound?

Yes

Are the conclusions drawn adequately supported by the results?

Yes

Are sufficient details of methods and analysis provided to allow replication by others?

Yes

Reviewer Expertise:

Behaviour science

I confirm that I have read this submission and believe that I have an appropriate level of expertise to confirm that it is of an acceptable scientific standard.

# Response

**Published:** 2024-04-08

**Authors:** Marta Marques

**Affiliations:** NOVA National School of Public Health, Lisbon, Portugal

We thank the reviewer for their positive feedback about this paper. All comments made by the reviewer have been addressed and implemented where appropriate.

**1. Figures**: Figure 1 and Figure 2 could be combined, because they describe the same process. Figure 2 was somewhat inconsistent in its presentation; e.g., sometimes the number of BCTs at the end of each step was mentioned, and sometimes it was omitted. Figure 2 had some minor spelling errors (e.g., emended instead of amended). Response: We have removed Figure 1 to reduce redundancy, as the steps are detailed in-text. We have also reviewed the presentation of Figure 2 and have revised it so that each step is consistent. We have also fixed the spelling errors. 

**2. Examples**: when authors provided examples, it was much easier to understand each element of the process from initial BCTT to final BCTO. However, the inclusion of examples was inconsistent, e.g., for Step 2, an example was provided where 19 BCTs were split, but no other examples were provided for the other actions at this step. It did seem as though the goal directed BCTs was used throughout the process as an example where they were relevant, including in Table 3, but when the goal directed BCTs were not relevant no example was provided. Response: We have now provided a number of examples throughout steps 2-5, as suggested. We hope these added examples are helpful to show how BCTs were transformed from BCTTv1 to BCTO.

**3. Table 1 - Glossary**: I appreciate the inclusion of a glossary to assist readers in unfamiliar terms, but I was confused by the terms that were included (and omitted) from the glossary. For example, the elements that comprise an ontology were included (e.g., entity, class, relationship, ontology), but also common software tools/platforms (e.g., EPPI-Reviewer and GitHub). I didn’t understand why both ‘class’ and ‘parent class’ were included. ‘Ontology’ was defined, but ‘taxonomy’ was omitted. Finally, the term ‘BCI content’ was defined, but the acronym BCI was not spelled out in the definition. I suggest a review of the glossary to ensure it is as useful as possible for readers. Response: The glossary was intended to include low frequency terms or terms that may be unfamiliar to a behavioural and social science audience (e.g. terms relating to ontologies). Both ‘class’ and ‘parent class’ are included as they refer different things. ‘Classes’ in ontologies represent types of entities (‘things’) in the world. They can be arranged hierarchically by the specification of parent and child classes. A ‘parent class’ is a class within an ontology that is hierarchically related to one or more child classes such that all members of the child class are also members of the parent class, and all properties of the parent class are also properties of the child class. We have made the following amendments to the glossary:

- Spelling out and defining ‘BCI’: A behaviour change intervention (BCI) is defined as “An intervention that has the aim of influencing human behaviour.” (Michie et al. 2021)
- Defining taxonomy: A taxonomy is defined as “A hierarchy consisting of terms denoting types (entities) which are linked by subtype relations.” (Arp et al. 2015)

**4. Table 3 - Excerpt from the BCTO**: I thought this excerpt helped to communicate the hierarchical relationship between the entities in the ontology. However, I could imagine it being even more useful if presented as a hierarchical figure, such as a tree diagram. This would more explicitly show the relationships between entities (e.g., through branches on the tree). A separate comment for Table 3 is that the inclusion of the BCIO codes (e.g., BCIO:007001) is somewhat confusing because that the table purports to be an excerpt of the BCTO, not the BCIO. In the Discussion, I do see that the BCTO will form part of the Behaviour Change Intervention Ontology (BCIO), so all BCTO entities would therefore have a BCIO code. However, it may assist readers include a clarifying note for the table, or omit the BCIO code. Response: We have now included a diagram/figure showing the relationships between entities for the ‘goal directed BCT’ group. This was generated via the dedicated online, open-access tool, BCIOVisualise ( https://bciovis.hbcptools.org/). We hope this is helpful for readers to visualise the hierarchical relationship between entities. As the visualisation of large, complex ontologies can be challenging, we have only included a snapshot (i.e. of the ‘goal directed BCT’ group), rather than the entire BCTO. For the BCIO unique IDs (e.g., BCIO:007001), you are correct that the BCTO forms part of the larger BCIO, which is why all entity URIs within BCIO (including BCTs, mode of delivery, mechanism of action ontologies etc.) begin with “BCIO”. To avoid confusion, we have added the following note for the table: “The BCTO forms part of the larger BCIO, therefore all entity URIs hold the form BCIO:XXXXXX” 

**5. Clarifications for non-expert readers**: the use of Box 1 to answer or clarify some questions that arose during the development process for the BCTO was very helpful. Two other clarifications - already described in text but useful to highlight - could clarify questions for interested, non-expert readers.

- The first clarification is about the difference between a taxonomy and an ontology, and why an ontology was needed even though the BCTT already exists and is widely used. This is already addressed in the introduction, but making this comparison more prominent could help a reader who knows about the BCTT, but not the BCTO and its advantages.

Response: We have addressed this in the Discussion (see section ‘Key differences between the BCTO and the Behaviour Change Technique Taxonomy v1 (BCTTv1)), however, we have added this point to Box 1 to highlight the following to readers : (i) the difference between a taxonomy and ontology, (ii) why an ontology is needed even though BCTTv1 exists, and (iii) advantages of BCTO over BCTTv1.

  

- The second clarification is about the difference between a behaviour change *technique *and a behaviour change *intervention*. This is mentioned several times in the introduction (e.g., “Behaviour change techniques are defined as…” and later “ontologies… can link BCTs to other intervention features such as their delivery…”) but again, making this comparison more prominent could help a reader understand how BCTs ‘fit’ within an intervention, and how the BCTO fits with the larger BCIO.

Response: We added definitions of ‘behaviour change technique’ and ‘behaviour change intervention’ to the Glossary. We have also added an overview of the larger BCIO to help readers visualise how behaviour change techniques form part of behaviour change intervention content (see Figure 1).
